# Cost per insertion and couple year of protection for postpartum intrauterine devices and implants provided during service scale-up in Kigali, Rwanda

**DOI:** 10.12688/gatesopenres.12858.4

**Published:** 2020-02-28

**Authors:** Kristin M. Wall, Rosine Ingabire, Susan Allen, Etienne Karita

**Affiliations:** 1 Department of Epidemiology, Rollins School of Public Health, Laney Graduate School, Emory University, Atlanta, GA, 30322, USA; 2 Rwanda Zambia HIV Research Group, Department of Pathology & Laboratory Medicine, School of Medicine, Rollins School of Public Health, Emory University, Atlanta, GA, 30322, USA; 3 Projet San Francisco, Rwanda Zambia HIV Research Group, Department of Pathology & Laboratory Medicine, School of Medicine, Rollins School of Public Health, Emory University, Kigali, Rwanda

**Keywords:** Couple year of protection, post-partum, intrauterine device, contraceptive implant, Rwanda

## Abstract

**Introduction:** In two high-volume government hospitals, their two affiliated health facilities, and two additional health facilities, we developed and implemented postpartum intrauterine device (PPIUD) and postpartum (PP) implant promotional counseling and service delivery procedures between May-July 2017 in Kigali, Rwanda. Between August 2017 and July 2018, 9,073 pregnant women received PPIUD/PP implant promotions who later delivered in one of our selected facilities. Of those, 2,633 had PPIUDs inserted, and 955 had PP implants inserted. The goal of the present analysis is to detail implementation expenditures and estimate incremental costs per insertion and couple years of protection (CYP) for PPIUD and PP implant users.

**Methods:** We detail the incremental costs during the implementation from the health system perspective (including both the implementation costs and the cost of contraceptive methods) and use of standard methods to estimate the cost per insertion and CYP for PPIUD and PP implant users. In addition to the incremental costs of labor and supplies, the costs of promotional activities are included. Research costs for formative work were excluded.

**Results:** A total of $74,147 USD was spent on the implementation between August 2017 and July 2018. The largest expense (34% of total expenses) went toward personnel, including doctoral-level, administrative, data management and nurse counseling staff. Training for PPIUD and implant providers and promoters comprised 8% of total expenses. Recruitment and reimbursements comprised 6% of expenses. Costs of implants to the government comprised 12% of the expenses, much higher than the cost of IUDs (1%). Costs per insertion were $25/PPIUDs and $77/PP implant. Costs per CYP were $6/PPIUDs and $21/PP implant.

**Conclusion:** Understanding the cost per PPIUD/PP implant inserted and CYP can help to inform the cost of scaling up PPIUD/PP implant service implementation activities and resource allocation decision-making by the Rwandan Ministry of Health.

## Introduction

Voluntary family planning (FP) is one of the most cost-effective public health interventions, reducing both maternal and child mortality and improving national economies ^1^. Postpartum FP in particular is critical to improve maternal-child health via birth limiting and spacing ^2^. However, there is high unmet need for family planning in the developing world, especially in postpartum periods. In postpartum periods, 61% of women across 21 low- and middle-income countries experienced unmet need ^3^, while 95% of women across 5 countries desired to avoid pregnancy for at least 1 year after delivery ^4^. In Rwanda, although only 2% of postpartum women report a desire for another child within 2 years of delivery, the unmet need in the postpartum period is 51% ^5^. The authors of this analysis defined unmet need for family planning prospectively as a less biased means to predict women’s family planning need in the postpartum period. This measure comprised assessment of women’s fertility preferences, timing of any desired subsequent pregnancy, and current contraceptive method use ^5^.

To meet women’s postpartum fertility goals and improve maternal-child health via birth spacing or limiting ^6^, the Rwandan government has made postpartum family planning a key objective of the Rwandan Family Planning 2020 Commitment (Objective 2: ‘Scale up the postpartum family planning (PPFP) in all health facilities in Rwanda to increase method choice including access to long term methods…’) with the goal of preventing 250,000 unintended pregnancies annually ^7^. There is a sizable opportunity to provide PPFP counseling or services among women at timepoints during which they are already engaged in the healthcare system such as during antenatal care (ANC), labor and delivery, and infant vaccination.

Long-acting reversible contraceptive (LARC) methods (the hormonal and copper intrauterine device (IUD) and hormonal implant) are not only the most effective reversible methods (lasting 5–10 and 3–5 years, respectively, with typical use failure rates <1%/year), but are very cost-effective ^8– 12^. A copper postpartum IUD (PPIUD) can be inserted immediately after delivery of the placenta, during a cesarean delivery, up to 48 hours after childbirth, or beginning at 4 weeks after delivery ^13, 14^ ( https://www.mcsprogram.org/resource/pathway-of-opportunities-for-postpartum-women-to-adopt-family-planning/). A postpartum (PP) implant can be inserted any time after delivery ( https://www.mcsprogram.org/resource/pathway-of-opportunities-for-postpartum-women-to-adopt-family-planning/). and the WHO Medical Eligibility Criteria were recently revised for postpartum implants ^15^. IUDs make up a relatively small share of method use in Rwanda (2.5% of the method mix), while implants make up 16.9% of the method mix ( http://www.familyplanning2020.org/entities/81).

This relatively low uptake is thought to be related to lack of method promotions to both women and their male partners as well as limited provider comfort counseling on and delivering these methods ^16– 20^. Because baseline knowledge about the LARC methods among potential users is lower than for other methods ^21– 25^, demand creation strategies must include comprehensive information addressing method benefits, side-effects, and misconceptions ^21, 26, 27^. To address these issues, funding from a Bill and Melinda Gates Grand Challenge Award was received to improve PPIUD supply and demand in Kigali, Rwanda, with supplementary funding from Emory University to provide PP implant services. Briefly, in two large health centers (providing ANC, family planning, and infant vaccination services), their two adjoining referral hospitals (providing routine and complex labor and delivery), and two additional large health centers (providing ANC, family planning, routine labor and delivery, and infant vaccination services), Emory-based non-governmental organization Projet San Francisco (PSF) developed and implemented PPIUD and PP implant promotional counseling and service delivery procedures in August 2017. The PPIUD and PP implant were promoted during ANC and labor and delivery to target women prior to delivery. Promotions also occurred during infant vaccination visits which have been shown to be an acceptable and high-impact venue to reach postpartum women in Rwanda ^28^ and are considered a potentially high-impact target for integration since immunization services have broad reach ^29^. By July 2018, 9,073 pregnant women received PPIUD/PP implant promotions who later delivered in one of our selected facilities. Of those, 2,633 had PPIUDs inserted, and 955 had PP implants inserted. These published findings represented a significant increase in PPIUD and PP implant uptake versus the 6 months prior to our implementation (p<0.001) ^30^.

The goal of the present analysis is to detail expenditures during the implementation and estimate the incremental cost per PPIUD insertion, PP implant insertion, and couple years of protection (CYP) for PPIUD and PP implant users to inform decision-making by the Ministry of Health and to estimate the cost of scaling up activities. Importantly, in addition to the costs of labor and supplies, the costs of promotional activities are included when calculating the costs and cost-effectiveness estimates of this intervention because postpartum LARCs are still relatively unknown (this is especially true for the IUD for which baseline knowledge is low ^21– 25^) and require a significant investment in demand creation.

## Methods

### PPIUD/PP Implant program development and operations

The PPIUD/PP implant intervention (described in detail previously ^30^) was developed with input from stakeholders, providers, community health workers (CHW), and couples/clients. Stakeholders included the Rwanda Ministry of Health, the District Mayors, the Rwandan Family Planning Technical Working Group, and clinic directors. Through formative work between May and July 2017, we evaluated knowledge, attitudes, and practices regarding PPIUD/PP implant services among community health workers and providers and clients/couples. This formative work led to the development of intervention operational procedures and a promotional counseling flipchart to be delivered to women or couples. Promotional counseling was conducted primarily by counselors during ANC, labor and delivery, and infant vaccination services or within the community by CHW. In addition, dedicated promoters were hired to administer promotions. In August of 2017, nurses and midwives working in labor and delivery and family planning departments began training in PPIUD insertions (implant insertion training had been previously provided). Clinic staff and CHWs were trained to promote the PPIUD/PP implant services. Follow-up appointments were scheduled for PPIUD clients within 6 weeks after PPIUD insertion (typically coinciding with the 6-week infant vaccination visit).

Pre-intervention postpartum LARC services were conducted by two national PPIUD trainers located at two of our selected district hospitals. One of these national PPIUD trainers was collecting PPIUD insertion and follow-up data in a logbook specifically for PPIUD services. In the 6-months prior to our intervention (from February-July 2017), n=46 PPIUDs were inserted (average of 7.7 insertions/month) and n=182 PP implants were inserted (average of 30.0 insertions/month) in the selected health facilities. The percent increase comparing monthly PPIUD insertions between February-July 2017 to our intervention period of August 2017-July 2018 was 2,687% for PPIUD and 169% for PP implant.

### Incremental PPIUD/PP implant program costs

We used a standard, comprehensive micro-costing approach as recommended to calculate the incremental cost of the PPIUD/PP implant intervention from the health system perspective ^31^. Using standardized data collection tools, resource use data was collected from expenditure records, study case report forms, and interviews with program implementers. Costs of labor, promotions, and supplies are included as detailed below, and no research costs are included. Thus, the costs included are the incremental costs required to implement the promotional counseling and service delivery intervention above the minimal existing pre-intervention postpartum LARC services described above. Study coordinators and the nurse counselor were responsible for arranging training activities, organizing PPIUD certifications, scheduling providers across the hospitals and health centers, and other implementation logistics. The data manager was responsible for extracting and recording the government logbook data to enable monitoring of PPIUD uptake and occurrence of PPIUD side-effects (e.g., infections) and expulsions. We envision that these would be regular activities required to implement and monitor a large-scale implementation. Part-time salaries and fringe were provided for three Emory staff and the PSF Director. PSF-based personnel included a dedicated physician with part-time support from two project physicians, two study coordinators, a senior nurse counselor, a data manager, and two promotions managers.

Per diems were provided for trainees during training activities. Training costs included the costs of training providers to insert PPIUDs during a 2-day didactic training and mentored practical certification process, and the costs of training PPIUD/PP implant promotional agents. Field travel included travel for Emory-based staff and transportation for local staff. Field travel was required to transport staff to trainings (which would be recurring during future implementation stages) and the implementation clinics. Other field expenses included wire transfer fees, transcription and translation services, and meals during trainings. Transcription and translation services were required to produce implementation tools in two of the main languages spoken in Rwanda (Kinyarwanda and French).

Recruitment/reimbursement expenses began in February/March 2018 and included: PPIUD client transport reimbursement for follow-up visits ($2.29 United States Dollars [USD]/client), reimbursements for CHW promoters ($0.57 USD/client presenting their referral when requesting a PPIUD or PP implant), reimbursements for providers ($1.20 USD/PPIUD and $0.57 USD/PP implant insertion), and reimbursements to the selected facilities for administrative costs associated with implementing the PPIUD/PP implant program ($57 USD/facility/month). Reimbursements for providers were higher for the IUD versus the implant because IUD insertion required additional training and certification time and each IUD takes longer to insert versus an implant. CHW and clinic provider reimbursements used the Rwandan performance-based-financing (PBF) system as a guide ^32^. Reimbursements for providers included the cost of providers’ time/labor to provide insertions. This was provided to them in addition to their regular salary (the average monthly salary for family planning or labor and delivery nurses is $124-364 USD, depending on their education). Communications expenses included internet and phone airtime for staff. Field consumables/office supplies included specula, forceps, batteries, logbooks, chargers for tablets, PPIUD kits and various office supplies. Tablets were used to collect data from logbooks for quality assurance/control.

We also included the cost of methods (estimated from the prices incurred by the United Nations Population Fund (UNFPA) in 2015 of $0.37 USD per copper T380 IUD and $8.93 USD per Jadelle levonorgestrel rod implant ( http://mshpriceguide.org/en/home/), and converted to 2018 USD ($0.39 and $9.49 USD, respectively). Expenditures are reported by activity in 2018 USD.

Only implementation costs related to service provision were included (i.e., we did not include research costs for formative work conducted between May and July 2017). Thus, the expenses presented represent the frontline incremental costs required to implement the program between August 2017 and July 2018 from the health system perspective. No discounting of costs was performed given the short time horizon. We follow the Consolidated Health Economic Evaluation Reporting Standards ^31^.

### PPIUD/PP implant program outcomes

Outcomes of interest include the number of PPIUDs and PP implants inserted and the cumulative couple years of protection (CYP) for PPIUD and PP implant users. CYP is a commonly used estimate of the length of contraceptive protection against pregnancy provided per unit of that method and is estimated at 4.6 for the Copper T380 IUD and 3.8 CYP for Jadelle (5 year) implant ^33^ ( https://www.measureevaluation.org/prh/rh_indicators/family-planning/fp/cyp).

CYP for the contraceptive methods are calculated by adjusting to remove any ‘double coverage’ caused by overlap with post-partum LAM during our 1 year time horizon. Using Rwandan DHS data, an analysis of sexually active women found pregnancy risk increases from 12% during the first six months postpartum to 53% from 6–12 months (and then decreases to 48% from 12–24 months) ^5^. According to this data, during the first 2 years postpartum only 5% of women practice at least periodic abstinence and over 85% of women breastfeed ^5^. Per DHS and USAID data, we assumed that all women were conferred protection by LAM during the first 6 months postpartum and that no women were protected by LAM after 6 months; we also assumed that there was no ‘double coverage’ due to abstinence, which is only practiced periodically ^5^ ( https://www.fhi360.org/sites/default/files/media/documents/postpartum-family-planning-immunization-integration-rwanda-2013.pdf). The estimated CYP for LAM is 0.25 CYP per user ( https://www.measureevaluation.org/prh/rh_indicators/family-planning/fp/cyp). Thus, the adjusted CYP for implant in our study is 3.7 and the adjusted CYP for the IUD is 4.5. 

Using the incremental cost measures and outcomes of interest, we calculated the cost per PPIUD inserted, cost per PP implant inserted, cost per CYP for PPIUD users, and cost per CYP for PP implant users. No discounting of outcomes was performed given the short time horizon of the 12-month implementation.

### Ethical considerations and consent

The Emory University Institutional Review Board (IRB) and the Rwanda National Ethics Committee (RNEC) approved the research component of the project (IRB 00001497). Written informed consent was obtained from all participants prior to enrollment. The Emory University IRB determined the programmatic service delivery component of the project (PPIUD promotions and insertions performed in government clinics) was exempt from review.

## Results

Raw data for this study are available in Dataset 1 ^34^.

### Incremental PPIUD/PP implant program costs

Program costs are summarized in Table 1. A total of $74,147 USD was spent on the implementation between August 2017 and July 2018. The largest expense (34% of total expenses) went toward personnel, including doctoral-level (MD and PhD) staff, and administrative, data management and nurse counseling staff. Trainings for PPIUD and implant promotional counselors and PPIUD providers comprised 8% of total expenses. Recruitment and reimbursements comprised 6% of expenses. Costs of implants to the government comprised 12% of the expenses, much higher than the cost of IUDs (1%).

**Table 1.** Allocation of costs for the PPIUD/PP implant implementation by activity (August 2017–July 2018). Only direct costs included; all costs in 2018 USD.

Costs incurred by implementation team	USD	Percentage of total
Salaries and fringe: PSF and clinic staff	$25,051	34%
Salaries and fringe: Emory employees	$14,225	19%
Trainings	$6,099	8%
Field travel	$5,363	7%
Other field expenses	$5,820	8%
Recruitment/reimbursement	$4,510	6%
Communication	$1,427	2%
Field consumables/office supplies	$1,129	2%
Field facilities	$433	1%
Cost of methods		
Cost of implants *	$9,063	12%
Cost of IUDs *	$1,027	1%
**Total Expenses**	**$74,147**	

*$0.39/IUD and $9.49/implant (2018 USD). PPIUD, postpartum intrauterine device; PP, postpartum, IUD, intrauterine device; USD, United States Dollars.

### PPIUD/PP implant program outcomes

Program outcomes are summarized in Table 2. Costs per insertion were $25/PPIUDs and $77/PP implant. Costs per CYP were $6/PPIUDs and $21/PP implant.

**Table 2.** Outcomes of interest for the PPIUD/PP implant implementation (August 2017–July 2018). All costs in 2018 USD.

IUD outcomes	Value
PPIUDs inserted (N)	2,633
Cumulative CYP for PPIUD users *	11,783
Cost per PPIUD inserted	$25
Cost per CYP for PPIUD users	$6
Implant outcomes	
PP Implants inserted (N)	955
Cumulative CYP for PP implant users *	3,510
Cost per PP implant inserted	$77
Cost per CYP for PP implant users	$21

*Assumes CYP for IUD is 4.5 and for the Jadelle implant is 3.7. PPIUD, postpartum intrauterine device; PP, postpartum; CYP, couple years of protection; USD, United States Dollars.

## Discussion

Our implementation provided services at a cost per insertion of $25 and $77 for the PPIUD and PP implant, respectively, and CYP of $6 and $21 for the PPIUD and PP implant, respectively. Understanding the cost per PPIUD/PP implant inserted can help to inform decision-making by the Ministry of Health and to estimate the cost of scaling up PPIUD/PP implant service implementation activities. Since cost per CYP is a standard and commonly used measure, our estimates of cost of CYP also help the government to determine contraception funding priorities.

For comparison, in a previous study conducted in Rwanda, 478 PPIUDs were inserted over 15 months in 12 sites at an incremental cost of $95,004 USD. After amortization of training costs over three years, investigators estimated outcomes of $110/PPIUD inserted and $24/CYP for the PPIUD ^16^.

For context, as reported previously ^30^ and in an upcoming publication, of women to whom we promoted PPFP to who delivered at a study facility, 11% selected the implant and 29% the selected the IUD. The average age of women receiving the IUD was 28.3 (standard deviation=6.0) and the implant was 27.0 (standard deviation =5.6) and average parity of IUD and implant users was 2.4 (standard deviation =1.4) and 2.3 (standard deviation =1.3), respectively. Among those who selected the IUD, 6% were expulsed (of those, 60% had an IUD reinserted and 12% had an implant inserted), 1% were removed, and 0.4% experienced infection ^30^. No postpartum implant side-effects were noted. Patients perceptions of anxiety and pain were low for both methods (2/10 on a Likert scale for the implant and 1.8/10 for the IUD), and reported satisfaction was high (>9.5/10) ^30^. An improved understanding of differences in women who uptake the IUD versus implant (versus another method entirely) would be informative for future scale up.

Though few additional postpartum contraception studies exist for comparability, other studies (summarized in Table 3) have made estimates of method cost per CYP, though not specifically in postpartum periods. The World Bank estimated that the cost per CYP for reversible modern methods in Ethiopia, Uganda, Burkina Faso, and Cameroon was lowest for the IUD ($4.14-$23.35), while the costs per CYP for oral contraceptive pills (OCPs) ($17.00-$31.45) and implants and injectables ($19.84-$58.54) were much higher ^35^. Using data from 13 USAID tier one priority reproductive health countries and service delivery costs, researchers estimated that the cost per CYP was $1.37 for the copper IUD, $4.67 for Sino-Implant, $7.07 for DMPA, $6.88 for combined OCPs, and $4.06 for Jadelle ^36^. Finally, a study in Zambia estimated costs per CYP were $8.69 for the IUD and $15.15 for the implant ^37^. The wide range of cost per CYP estimates are related to variation across countries in costs of trainings; consumable supplies; instruments; and labor for counseling, insertion, removal, and resupply.

**Table 3.** Comparison of CYP for different contraceptive methods (not specifically postpartum) from select studies.

Method	Cost per CYP	Reference
Copper IUD	$1.37-$23.35	35– 37
Implant	$4.06-$15.15	36, 37
OCP	$6.88-$31.45	35, 36
DMPA injectables	$7.07	36
Implants and injectables (combined in four of the studies included)	$19.84-$58.54	35

CYP, couple years of protection; IUD, intrauterine device; OCP, oral contraceptive pill; DMPA, depot medroxyprogesterone acetate

The cost per CYP for the PPIUD and PP implant in our study were within the range of these other non-postpartum focused studies. Although it is difficult to compare estimates of cost per CYP across studies because of different approaches to measuring and including costs and because of the different implementation models used (for example, Neukom *et al.* ^37^ used a dedicated provider model whereas in our study PPIUD and PP implant services were provided by existing providers in addition to their regular duties with promotions being conducted by those providers and dedicated promoters), these studies indicate that the IUD has the lowest cost per CYP versus other reversible methods, and that estimated costs per CYP are generally higher for the implant versus the IUD, largely because of difference in commodity costs which is the main driver in the difference of the costs of these two methods ( http://mshpriceguide.org/en/home/).

Importantly, these studies did not include the cost of demand creation activities. The cost of promotional counseling activities is important for implementers to consider when evaluating postpartum and LARC-focused interventions because postpartum LARCs are still relatively unknown and require a significant investment in demand creation. Studies support that such promotional activities should also educate men ^38– 41^, as done in this study, which incurs additional costs above focusing promotional activities on women alone. Once social diffusion is achieved and the target population is knowledgeable about postpartum LARC methods, demand creation activities can decrease.

While the IUD was promoted in the context of the full range of method options, we dedicated more time to discussing the PPIUD because it is the least well-known method in sub-Saharan Africa, including in Rwanda ^22, 42– 45^, which explains the relatively high uptake of the IUD relative to the implant. Other LARC implementation studies have observed that the implant is more popular than the IUD ^46, 47^ but that this trend shifts after focused IUD educational and counseling efforts, community-based and media efforts, and provider refresher IUD trainings ^25, 48^. Thus, though the IUD is less well-known versus the implant in much of sub-Saharan Africa ^22, 42, 43, 45, 49^ and providers may have lower baseline comfort promoting and inserting IUDs ^18– 20^, concerted promotional counseling and training efforts can be successfully employed as was achieved in these examples and our study to increase IUD demand.

There is very limited literature on the cost of the PP implant, making our findings timely especially in light of the WHO Medical Eligibility Criteria (MEC) updates related to the postpartum implant ^15^. Women who are <6 weeks postpartum and breastfeeding can use the implant with a MEC of 2 (meaning the method is generally recommended) while all other women can use the implant regardless of breastfeeding with a MEC of 1 (meaning no restrictions on use). Given the preference for the implant observed in some studies ^46, 47^, if the commodity costs for implants were reduced this method could become even more affordable for health systems to scale-up.

### Limitations

Similar to the other studies cited here, we included costs from the health system perspective only; however, we recognize that more detailed costing analyses including the societal perspective including women’s time and the value of their time would be informative and may strengthen evidence to increase LARC services (since women are saved time traveling to clinic for OCP refills or 3-monthly injectables). It would have also been informative to estimate the cost per promotional method employed (e.g., promotions occurring during ANC, labor and delivery, infant vaccination, or delivered in the community by CHW), but as many women received multiple promotions from several places and our promotional strategies evolved over time, this was not possible in the present study. Given our short time horizon, we did not amortize our training costs as in another PPIUD/PP implant in Rwanda ^16^, though the education provided during trainings may translate into service provision over several years in the future; amortization would have decreased our estimated costs per insertion and CYP. We did not collect the data needed to divide the costs of consumables such as specula by their number of uses to arrive at per insertion costs. Additional supplies such as alcohol pads and gauze were among government supplies used and were not measured or included in our calculations. It is not certain whether the cost outcomes estimated here would apply directly larger scale-up activities. We hypothesize that economies of scale may be gained, for example when training a larger number of nurses simultaneously, but it remains to be seen whether quality services can be provided for the same (or reduced) CYP at scale. Finally, our results are most generalizable to sub-Saharan African countries. 

## Conclusions

There is consensus in the international community that greater investment in postpartum family planning, and the IUD in particular, is needed. We have developed a successful, multi-level intervention that increases PPIUD and PP implant uptake that has relatively low costs per insertion and CYP. Future analyses will explore whether the intervention is cost-effective (or potentially cost-saving).

## Data availability

Underlying data are available from Harvard Dataverse. Dataset 1: Replication Data for an interim evaluation of a multi-level intervention to improve postpartum intrauterine device (PPIUD) services in Rwanda ( https://doi.org/10.7910/DVN/WLZ7PC) ^34^.

Data are available under a Creative Commons Zero (“CC0”) Public Domain Dedication Waiver.
