# Supplementary material for: Cost per insertion and couple year of protection for postpartum intrauterine devices and implants provided during service scale-up in Kigali, Rwanda
Source: Gates Open Res. 2020 Feb 28;2:39. Originally published 2018 Aug 31. [Version 4] doi: 10.12688/gatesopenres.12858.4 (PMC7163922; doi:10.12688/gatesopenres.12858.4)
Supplement: Peer review history

# Peer review: Reviewer response for version 4

**DOI:** 10.21956/gatesopenres.14293.r28636

**Published:** 2020-04-14

**Authors:** Kate H. Rademacher

**Affiliations:** 1 FHI 360, Durham, NC, USA

Thank you for addressing these comments and for your thoughtful responses.

I confirm that I have read this submission and believe that I have an appropriate level of expertise to confirm that it is of an acceptable scientific standard.

# Peer review: Reviewer response for version 3

**DOI:** 10.21956/gatesopenres.14061.r27631

**Published:** 2019-09-09

**Authors:** Aisha N.Z. Dasgupta

**Affiliations:** 1 United Nations Population Division, New York, NY, USA

Thank you for the opportunity to review this paper, which provides useful insights into cost per method, and cost per CYP, for postpartum IUD/implants. It is a helpful contribution to the literature because of the evidence around value for money of such projects. I recommend Approval with reservations.

 Introduction:

- In the first paragraph, I was at times a little confused when you write about unmet need during the postpartum period. Is this about need at that particular point in time based on prospective fertility intentions, or were women determined to have unmet need based on their retrospective fertility intentions regarding their current pregnancy/most recent birth? For example, “In Rwanda, although only 2% of postpartum women report a desire for another child within 2 years of delivery, the unmet need in the postpartum period is 51%”. Is this 51% based on prospective fertility intentions which suggests a current unmet need? Or is some of this 51% based on the retrospective fertility intentions regarding the most recent birth? This is important to know as it influences how to interpret such a figure. If a notable portion of the 51% is based on retrospective fertility intentions, then it does not necessarily follow that postpartum family planning services is the answer to addressing high levels of unmet need, as these postpartum women might not be currently at risk of unintended pregnancy. Instead it could point to a general failure of services that lead to women having an unintended pregnancy (not necessarily during the postpartum period) who then appear in the unmet need estimate simply based on their retrospective reports of fertility intentions. I think it is important to know, because it helps us to understand exposure to unintended pregnancy during the postpartum period (I come back to this again later), and justifies the interest in the postpartum period and any related postpartum FP interventions.
- Related to this, perhaps the authors could point to other reasons why there is interest in interventions during the postpartum period. E.g. an opportunity to engage with and target women as they are typically already in contact with the health care system for themselves and their infants (ANC, labor/delivery, vaccinations etc.).
- A general comment for the paper: My main reservation is there is no mention of the risk of pregnancy in this population during the postpartum period, and the implications for the analysis. For example, I would like to know a little more information about any practice of postpartum abstinence, and how long is postpartum amenorrhea? What are the breastfeeding practices in this population? It would be possible to provide this type of information on duration of postpartum insusceptibility from the Demographic and Health Survey and perhaps other sources. I took a quick look at the 2014-2015 DHS and it looks like women are either still amenorrheic or still abstaining for around one year.  
- As the authors explain, a strength of LARCs is they can provide protection for 5-10 (IUD) and 3-5 (implant) years, and the CYP is 4.6 for IUD and 3.8 for implant. But, is it fair to claim full CYPs in this analysis, when it is known that the population being provided with IUDs and implants are on average at lower risk of pregnancy, at least for around the first year postpartum? This is a limitation of the analysis and should be acknowledged in the discussion.

 

 Methods:

- Could you comment on the reimbursements to promoters and providers and why it was different for the two methods? What kinds of incentives did this create and could it have influenced which method was being recommended to potential clients?

 

 Results:

- There is an impressive number of study participants. Could you give any information on number of refusals to participate in the study? Is there any information on IUD/implant removals/discontinuation, side-effects, dissatisfaction?
- The results section seems a little thin. I was curious to know anything about the clients that were reached – is there any demographic information – age, marital status, education, wealth – anything? Were there any differences between implant/IUD users, in terms of background characteristics? This might help the reader to understand the value for money in terms of the two methods, particularly if one method was more popular with a hard-to-reach group.
- Could you provide a little more understanding of why implants were more expensive? Is this only because the commodity is more expensive, or were there other factors as well? Would the cost per PP implant have been lower, if a larger number of clients had been reached?

 

 Discussion:

- I note in Table 3 such a wide range of estimates of cost per CYP for IUD, which is quite hard to interpret. In Table 3, should the title of the table and/or the column title be “cost per CYP”, rather than “CYP”? Perhaps you could also provide a column providing the CYP itself for each of these methods.
- “ **The CYP** for the PPIUD and PP implant in our study were within the range of these other non-postpartum focused studies.” Do you mean the “cost per CYP”?
- I appreciate your second-to-last paragraph in the discussion, on the possibility of IUDs in sub-Saharan Africa.

I confirm that I have read this submission and believe that I have an appropriate level of expertise to confirm that it is of an acceptable scientific standard, however I have significant reservations, as outlined above.

# Response

**Published:** 2020-02-26

**Authors:** Kristin Wall

We appreciate these thoughtful comments which have further improved the manuscript.

In the introduction, we clarify that the estimate of 51% unmet need is based on a prospective assessment as a less biased means to predict women’s family planning need in the postpartum period. We now highlight in the introduction the sizeable opportunity to provide PPFP counseling/services at timepoints when women are already engaged in the healthcare system. The CYP for the contraceptive methods are now calculated adjusting to remove any ‘double coverage’ caused by overlap with post-partum insusceptibility, and we discuss changes in postpartum pregnancy risk as well as frequency of breastfeeding, LAM, and periodic abstinence in this population. Reimbursements for providers were higher for the IUD versus the implant because IUD insertion required additional training and certification time and each IUD takes longer to insert versus an implant. For further context, we now describe (as reported previously and in an upcoming publication), of women to whom we promoted PPFP to who delivered at a study facility, 11% selected the implant and 29% the selected the IUD. The average age and parity of women selecting the IUD or implant, as well as frequency of removals, side-effects, pain/anxiety during insertion, and satisfaction are now reported. An improved understanding of differences in women who uptake the IUD versus implant (versus another method entirely) would be informative for future scale-up, as now mentioned. We clarify that the driver of the difference in implant versus IUD costs are commodity costs. The wide range of cost per CYP estimates from other studies are related to variation across countries in costs of trainings; consumable supplies; instruments; and labor for counseling, insertion, removal, and resupply.

# Peer review: Reviewer response for version 2

**DOI:** 10.21956/gatesopenres.14020.r26919

**Published:** 2019-03-19

**Authors:** Kate H. Rademacher

**Affiliations:** 1 FHI 360, Durham, NC, USA

The authors made substantial improvements in this version of the paper. Thank you for revising.

 As part of this, thank you for clarifying that the cost analysis described in the paper did not include the costs of implementing the research. However, I am still confused why salaries for “study coordinators” and “data managers” were included – these sound like they were costs associated with research. Please clarify, and revise analysis if needed.

 In addition, here are some other more minor comments:

- Introduction – consider a transition sentence between the first two sentences – e.g. discuss the benefits of PPFP. Also, in the third sentence, please clarify that citation #3 refers to a study that took place in 5 countries (not the 21 that you referenced earlier in the sentence – citation #2).   
- Introduction – most hormonal IUD products are registered for 5 years duration. Please clarify in text.
- Introduction – you state that IUDs and implants make up “a relatively small share of method use in Rwanda.” However, I’m not sure it’s fair to lump these two methods together as 17% for implants seems like a relatively high proportion. Rwanda was not included in the analysis in this recent paper by Jacobstein (2018 ^1^), but still it would be good to more fully acknowledge the growing popularity and use of implants in SSA.
- Note some minor typos – e.g. name of Project San Francisco – “project” is misspelled.
- Discussion section – I would include mention of this study among PP women in Rwanda: Dulli *et al.* (2016 ^2^). Related to this suggestion, it sounds like promotion of PPIUD and PP implants in your intervention occurred in part in clinics which offer child immunization services. You may want to highlight this because FP-Immunization integration is considered a “promising” High Impact Practice by USAID, UFNPA and others. See here: https://www.fphighimpactpractices.org/briefs/family-planning-and-immunization-integration/.

- Discussion – you noted that in your intervention you “focused your promotions on the IUD” – please clarify the meaning here, as it is important to clarify if the IUD was promoted in the context of full method choice.

I confirm that I have read this submission and believe that I have an appropriate level of expertise to confirm that it is of an acceptable scientific standard, however I have significant reservations, as outlined above.

## References

[1] : Liftoff: The Blossoming of Contraceptive Implant Use in Africa. *Global Health: Science and Practice*.2018; Reference source 10.9745/GHSP-D-17-00396PMC587807029559495

[2] : Meeting Postpartum Women's Family Planning Needs Through Integrated Family Planning and Immunization Services: Results of a Cluster-Randomized Controlled Trial in Rwanda. *Glob Health Sci Pract*.2016;4(1) : 10.9745/GHSP-D-15-00291 73-86 10.9745/GHSP-D-15-00291 27016545PMC4807750

# Peer review: Reviewer response for version 1

**DOI:** 10.21956/gatesopenres.13941.r26758

**Published:** 2018-12-17

**Authors:** Kate H. Rademacher

**Affiliations:** 1 FHI 360, Durham, NC, USA

Postpartum family planning is a very timely and important topic, and I appreciate the authors efforts to conduct a costing analysis of the PPIUD and PP-implant intervention described. However, I have substantial concerns about the methods described, and therefore am not recommending indexing of the article in its current format. I encourage the authors to consider re-doing the costing analysis to incorporate the feedback below.

 A summary of my concerns about the methods are as follows:

1. The authors say that they use “a standard, comprehensive micro-costing approach as recommended to calculate the net cost of the PPIUD/PP implant intervention from the payer perspective.” They reference the CHEERS economic standards described in the paper by Husereau et al. However, in the paper by Husereau et al, there is no recommendation for calculating “net costs” which is what the authors say they did. Instead, in the CHEERS checklist in the Husereau et al. paper, they indicate that * incremental* costs should be calculated/reported. The reporting of "net costs" begs the question net of what? I encourage the authors to re-do the analysis and report incremental costs of the intervention (see comment #2 below for more on this).
2. The biggest concern I have about the methods is that it appears that research costs were included in the cost calculation. This is not typical in a micro-costing analysis like this. Typically, only the incremental costs of the intervention itself are included in a costing analysis (unless the authors expect the research component to continue as part of the standard way that the service is provided moving forward). As such, I would not include costs in your analysis such as salaries for researchers, salaries for study coordinators, travel for research staff, tablets (if used for data collection), transcription of research interviews, etc.
3. In addition to the issues noted above, the authors indicate that they are reporting from the “payer” perspective but they do not indicate who the “payer” is. The client? The health system? This is important because some costs that were included in the analysis should potentially be removed depending on who the payer is. For example, if you’re reporting costs from the health system perspective, then the “client transport reimbursement for follow-up visits” should not be included if that is a cost only incurred by the woman.
4. A smaller comment: please also indicate if costs of consumables/ supplies (such as specula, forceps) were divided by an estimated number of uses to get a per insertion cost. 
5. Related to #2 above, the authors conclude that the results can “inform decision-making…to estimate the cost of scaling up activities.” But is unclear if/how the intervention costs would differ from scale-up costs. It would be helpful to know which components of the intervention, if any, were only included in the pilot phase vs. would be included in potential later scale-up phase(s).
6. The authors make the statement that “The PPIUD/PP implant service implementation provided services at a low cost per insertion and CYP.”. However, it not clear what comparison is used to draw this conclusion. Low compared to what? In the Discussion section, the authors compare the costs to another PPIUD study in Rwanda and indeed, the cost per insertion and cost per CYP of the PPIUD was lower in the intervention described in this paper than in the previous anlaysis. But what about the cost of the PP-implant described in this paper? That cost is relatively quite high compared to the PPIUD and the authors do not comment on that. Other data from the World Bank and another paper by Tumlinson et al also cited, but it is not clear which of these data are being used for the comparison to the results described in this paper. One potential take-away is that the cost of the PP-IUD is much lower the cost of PP-implants. There is not much in the literature about the cost of provision of PP-implants given that the WHO Medical Eligibility Criteria (MEC) was recently updated regarding PP provision of implant, so a discussion about this would be a valuable addition to the literature.  

Additional smaller comments:

- In the first paragraph of the introduction, the following statement is made: “In post-partum periods, 50–90% of women experience unmet need, while 95% of women desire to avoid pregnancy for at least 1 year after delivery.” However, the research that is cited (Pasha et al.) only includes data from five countries whereas the statement the authors make is very broad. Consider revising to clarify. Also, see paper with analysis of data from 21 countries by Moore Z *et al*. (2015) ^1^.
- Introduction – authors should acknowledge that WHO MEC was recently updated regarding PP insertion of implants ^2^.
- In the third paragraph of the Introduction– the category of LARCs also includes the hormonal IUD (the LNG-IUS). Suggest revision to acknowledge this; it could just be a footnote.
- Introduction – citation 7 – This is a white paper form 2005. there are many more recent papers that document the effectiveness and cost-effectiveness of FP methods. Suggest replacing with a more recent citation.
- Note that the analysis in the paper in citation #18 by Tumlinson et al was updated in a more recent paper. I encourage you replacing the data/citation here this this more recent work. See: Rademacher *et al.* (2016) ^3^.
- In the Ethics section, it was confusing how the focus group discussions and surveys related to the costing component, if at all. Consider revising to clarify.
- I realize that you describe the intervention in-depth in another paper, but I suggest that you clarify in this article what the baseline service delivery model was for PPFP at the sites before the intervention was introduced. That will allow you describe and calculate the incremental costs of the new PPIUD and PP-implant intervention being evaluated.
- The Neukom et al paper that is cited (Citation #18) used a dedicated provider model. Related to point above, it would be good to clarify if dedicated providers were employed as part of the intervention, or it PPIUD and PP-implant were added to the scope of work for existing providers at the facilities. If it was the latter, this would not be a fair comparison to the costs described in the Neukom et al paper as the models are different. And again, the key is to calculate the incremental costs of the intervention you described – so only the incremental costs of having providers add PPIUD/PP-implants to their service package.

 

 Thank you for the chance to review. Again, this is an important topic, and I hope the authors will consider revising and re-submitting.

I confirm that I have read this submission and believe that I have an appropriate level of expertise to state that I do not consider it to be of an acceptable scientific standard, for reasons outlined above.

## References

[1] : Missed opportunities for family planning: an analysis of pregnancy risk and contraceptive method use among postpartum women in 21 low- and middle-income countries. *Contraception*.2015;92(1) : 10.1016/j.contraception.2015.03.007 31-9 10.1016/j.contraception.2015.03.007 25769442

[2] Medical eligibility criteria for contraceptive use: Fifth Edition. *World Health Organisation*.2015;26447268

[3] : Expanding Access to a New, More Affordable Levonorgestrel Intrauterine System in Kenya: Service Delivery Costs Compared With Other Contraceptive Methods and Perspectives of Key Opinion Leaders. *Global Health: Science and Practice*.2016;4(Supplement 2) : 10.9745/GHSP-D-15-00327 S83-S93 10.9745/GHSP-D-15-00327 PMC499016527540128

# Peer review: Reviewer response for version 1

**DOI:** 10.21956/gatesopenres.13941.r26628

**Published:** 2018-09-03

**Authors:** Katherine Tumlinson

**Affiliations:** 1 Department of Maternal and Child Health, Gillings School of Global Public Health, University of North Carolina at Chapel Hill, Chapel Hill, NC, USA

**Overall**: This is a well-written paper that makes a valuable contribution to the current body of literature by describing the cost per couple year of protection of LARC methods in Rwanda, after factoring in the cost of activities/materials designed to increase demand for these methods.

 I would encourage the authors to be more explicit about why such promotional activities are necessary for LARCs and why it is important to factor in the cost of promotional/demand-creation activities when calculating CYP for LARCs.

 I would also encourage the authors to include the cost of labor and supplies required for LARC insertion in their calculation of LARC CYPs (or to make this more explicit if they have already done so).

 Additionally, in the discussion section, I would encourage the authors to present the data comparing the CYP of other methods in a visual format so that readers can more easily interpret the results presented in this paper relative to the CYPs of shorter-acting methods or other LARC + promotion CYP calculations.

 These recommendations are described in more detail below, along with more minor suggestions. Once these concerns are addressed, I strongly recommend indexing. Thank you for the opportunity to review this paper.

**Abstract**:

1. The introduction presents the results of the parent study/intervention which was designed to increase demand for LARCs within a small number of facilities in Kigali. I encourage the authors to re-write this paragraph to better lay the foundation for the specific goal of this current paper. The authors may want to consider a short statement of the high unmet need during the postpartum period and the low prevalence of LARCs and the value of better understanding CYP for LARCs after factoring in the cost of promotional/demand-creation activities.
2. Similarly, the methods section of the abstract falls a little bit short; it would be helpful to indicate that the authors utilized a standardized method for calculating net cost of the intervention.

**Introduction**:

1. Overall the introduction is very well written and pleasantly concise. However, I would recommend insertion of a short paragraph that helps the readers to understand a key challenge of LARCs: promotional or demand creation activities are often necessary to increase uptake. Few prior studies (to my knowledge) have been able to calculate a CYP for a LARC that includes the cost of these demand creation activities. This is an important strength of this paper and should be highlighted.
2. An additional recommendation for the introduction involves the description of the parent study/intervention. In the second sentence of paragraph four, the authors briefly describe the results of the parent study in terms of the enormous increase in uptake. This is important information; however, it feels as if the authors have cut and paste from the abstract of the prior paper and – on first glance – it was confusing as I didn’t realize the authors were describing the intervention study and I mistakenly thought the results of the current paper were being summarized in the introduction. I recommend revisions so that this paragraph does a better job of explaining that there was a parent study/intervention that was found to be enormously successful in increasing the uptake of LARCs in select Kigali facilities and now the authors are writing this paper with the goal of understanding the CYP of LARCs, factoring in the cost of these very effective promotional activities.

**Methods**:

1. I would consider moving the first paragraph (ethics) to the end of the methods section, if possible. I was confused to read about focus group discussions in the first sentence, since I didn’t yet understand that formative research was done prior to designing effective promotional activities, neither of which are the real focus of this paper.
2. Are there available data on the cost of the supplies needed for insertion and the cost of provider’s time for insertion? For example, in a paper I wrote (which the authors cite, reference #17) that included CYP of various methods, we included the cost of supplies ($1.24) and labor (2.91) when calculating CYP for Sino-Implant ($12.10 total direct cost). I see the authors include “reimbursements for providers” but it’s not clear if this is the cost of labor (and, if so, it seems low). I also see consumables and supplies which appear to include specula and forceps, but I imagine there may be other supplies needed, for example alcohol pads, gauze/bandage, etc. It should not be difficult to obtain this data if not currently in hand.

**Results**: Excellent and concise presentation of exciting results!

**Discussion**: Again, well written and compelling. I have just a few suggestions for improvement:

1. The authors discuss data from a prior study in Rwanda and also recent World Bank data collected across multiple countries. Is it possible to present any of these data in a visual format alongside the results of this paper? It appears that the promotional approach used in the intervention study represents an improvement over prior efforts to increase LARC uptake in Rwanda – 3500 LARCs were inserted within one year in just 6 facilities and the total cost was under $75k. Can the authors create a graph that compares CYP from this study to the CYP from the FHI study so that readers can quickly see/digest that the CYP in this study was about one-fifth of the prior FHI study?
2. Can the authors present their calculated CYP for LARCs in a graph alongside the current CYP for shorter-term methods in Rwanda? This could help to highlight important differences in CYP across methods and make the case for larger investment in LARCs (or, at least, IUDs) as well as scaling up of the promotional flip-chart and training activities incorporated in the parent intervention.
3. When discussing the World Bank and USAID data, the authors should also indicate whether these prior studies included the cost of any demand generation activities (probably not).
4. In the last paragraph, prior to discussing limitations, the authors discuss the overall finding that IUDs represent significant cost savings over implants, largely due to the difference in the cost of the commodities. Some may argue, however, that IUDs represent a more difficult “sell” because they are more invasive (and painful?) to insert. Yet numerous women in the current study opted for the IUD over the Implant. Could the authors include data that might explain the comparative popularity of the IUD over the Implant among women in this study and discuss any implications?

I confirm that I have read this submission and believe that I have an appropriate level of expertise to confirm that it is of an acceptable scientific standard, however I have significant reservations, as outlined above.
